# Single plasmid systems for inducible dual protein expression and for CRISPR-Cas9/CRISPRi gene regulation in lactic acid bacterium *Lactococcus lactis*

**DOI:** 10.1038/s41598-018-19402-1

**Published:** 2018-01-17

**Authors:** Aleš Berlec, Katja Škrlec, Janja Kocjan, Maria Olenic, Borut Štrukelj

**Affiliations:** 1 Department of Biotechnology, Jožef Stefan Institute, Jamova 39, SI-1000 Ljubljana, Slovenia; 2 Graduate School of Biomedicine, Faculty of Medicine, University of Ljubljana, SI-1000 Ljubljana, Slovenia; 3 Faculty of Pharmacy, Charles University in Prague, 500 05 Hradec Králové, Czech Republic; 4 Faculty of Pharmacy, University of Ljubljana, Aškerčeva 7, SI-1000 Ljubljana, Slovenia

## Abstract

*Lactococcus lactis* is a food-grade lactic acid bacterium that is used in the dairy industry as a cell factory and as a host for recombinant protein expression. The nisin-controlled inducible expression (NICE) system is frequently applied in *L. lactis*; however new tools for its genetic modification are highly desirable. In this work NICE was adapted for dual protein expression. Plasmid pNZDual, that contains two nisin promoters and multiple cloning sites (MCSs), and pNZPolycist, that contains a single nisin promoter and two MCSs separated by the ribosome binding site, were constructed. Genes for the infrared fluorescent protein and for the human IgG-binding DARPin were cloned in all possible combinations to assess the protein yield. The dual promoter plasmid pNZDual enabled balanced expression of the two model proteins. It was exploited for the development of a single-plasmid inducible CRISPR-Cas9 system (pNZCRISPR) by using a nisin promoter, first to drive Cas9 expression and, secondly, to drive single guide RNA transcription. sgRNAs against *htrA* and *ermR* directed Cas9 against genomic or plasmid DNA and caused changes in bacterial growth and survival. Replacing Cas9 by dCas9 enabled CRISPR interference-mediated silencing of the *upp* gene. The present study introduces a new series of plasmids for advanced genetic modification of lactic acid bacterium *L*. *lactis*.

## Introduction

*Lactococcus lactis* is a gram-positive lactic acid bacterium that is used widely in the dairy industry^1^. In addition to that, it is becoming established as a microbial cell factory and as a host for recombinant protein expression. Its advantages are generally recognized as safe (GRAS) status and absence of endotoxins^2^. *L. lactis* was recently recognized as a probiotic^3^, and has been genetically engineered as a vector for the delivery of antigens and therapeutic proteins to the mucosal surfaces^4^. Tools for recombinant protein expression have been relatively well developed^2^. *L. lactis* is therefore comparable to other well established bacterial expression systems, such as *Escherichia coli* and *Bacillus subtilis*^2^.

The nisin-controlled expression system is probably the most commonly used for inducible expression in *L. lactis*^5–7^. Nisin is an antimicrobial peptide whose biosynthesis is controlled by a cluster of 11 genes. Among them, *nisR* and *nisK* encode transmembrane receptor NisK and the response regulator NisR, respectively. On binding nisin, NisK phosphorylates NisR, which in turn activates the nisin promoter PnisA. An example of the nisin-controlled expression system is the combination of *L. lactis* strain NZ9000, that contains *nisRK* in its genome, with plasmid pNZ8148, which enables cloning of the gene of interest downstream of PnisA^7^.

Advanced techniques for genetic engineering are required in order to develop *L. lactis* further as a microbial cell factory. Simultaneous expression of two or more proteins is beneficial for various applications, including the expression of multi-subunit proteins, the use of *L. lactis* as a mucosal delivery vehicle or as a multistep biocatalyst. Simultaneous inducible co-expression of multiple recombinant genes is established in the prototype bacterium *E. coli*^8–10^. Multiple proteins can be expressed from a single polycistronic vector containing a strong promoter, such as T7, and a series of genes separated by ribosome binding sites (RBSs)^10^. Polycistronic expression usually results in decreased yields of the protein that is further downstream from the promoter. This can be improved by the addition of an additional, strong promoter upstream of every recombinant gene^9^. Such plasmids are commercially available for *E. coli* (e. g. the pETDuet series)^11^, but are not readily available for *L. lactis*.

Clustered regularly interspaced short palindromic repeats (CRISPR)-Cas9 constitute an RNA-guided adaptive immune system of bacteria against invasive genetic elements^12^. CRISPR-Cas9 derived genome editing tools have revolutionized the fields of genetics, biology and biotechnology in model eukaryotic organisms^13,14^. Despite their bacterial origin, the use of CRISPR-Cas9 systems is less common in bacteria^13–16^. Nevertheless, their applications in bacteria include genome editing, gene regulation, production of next generation antimicrobials, DNA imaging, etc.^13,16,17^. Some of the most recent applications include multiplex genome editing^18^ and improved biosynthesis of chemicals^19^. CRISPR-Cas9 systems have been applied in various bacterial genera, including *Bacillus* and *Escherichia*, as well as non-traditional hosts *Clostridium, Streptococcus, Streptomyces, Mycobacterium*, and others^13^. Among lactic acid bacteria, CRISPR-Cas9 was applied in *Lactobacillus reuteri* for eradication of unmodified transformants following recombineering^20^ and for modification of the lactococcal phage genome^21^.

In the present work, plasmids for co-expression of two recombinant proteins in *L. lactis* have been developed and their effectiveness assessed by the expression of model proteins. Plasmids were further upgraded and a single plasmid CRISPR-Cas9 system has been developed. Plasmids will be applied in the future research in *L. lactis* for concomitant expression of therapeutic and reporter proteins, as well as for plasmid curing and gene silencing.

## Results

### Construction of plasmids for dual protein expression

**Figure 1:**
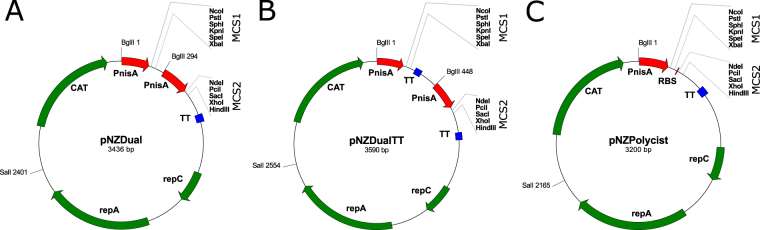
Schemes of plasmids pNZDual (**A**), pNZDualTT (**B**) and pNZPolycist (**C**) with depiction of multiple cloning sites MCS1 and MCS2. PnisA: nisin promoter. TT: transcription terminator. CAT: chloramphenicol acetyl transferase. RepA, RepC: replication proteins.

The dual promoter plasmid, pNZDual (Fig. 1A), for concomitant expression of two proteins, was obtained from pNZ8148. First, the NdeI site was removed from the backbone of pNZ8148 by site directed mutagenesis. PnisA was amplified from pNZ8148 by PCR and, at the same time, a second multiple cloning site, (MCS2), containing unique restriction recognition sites NdeI, PciI, SacI, XhoI and HindIII was introduced using appropriate primers. The transcription terminator sequence of pNZ8148 was cloned to pNZDual downstream of MCS1, resulting in pNZDualTT (Fig. 1B). In both pNZDual and pNZDualTT, the original transcription terminator of pNZ8148 downstream of MCS2 was retained. The single promoter plasmid, pNZPolycist, (Fig. 1C) for concomitant expression of two proteins from polycistronic RNA, was also obtained from pNZ8148. A gene fragment (53 bp) containing the ribosome binding site (RBS) and MCS2 and with unique restriction recognition sites was amplified with partially overlapping primers and cloned into pNZ8148m.

### Co-expression of IRFP and DARPin surface display cassette with plasmids for dual protein expression

**Figure 2:**
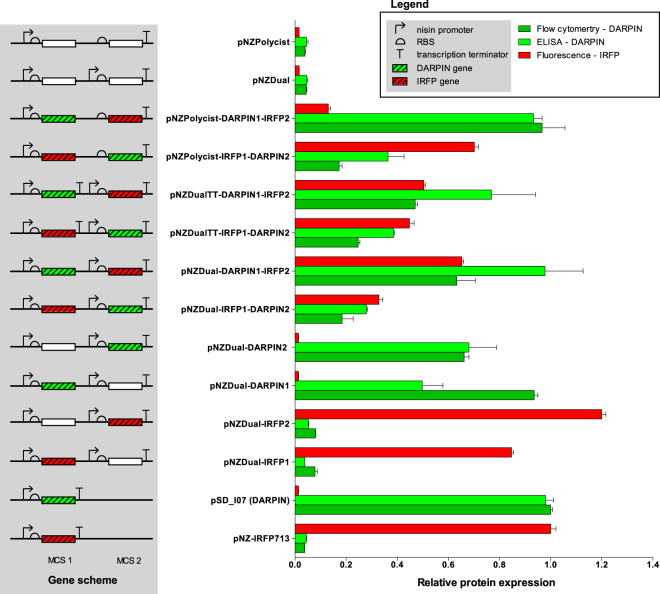
The expression of IRFP and DARPin surface display cassette genes, cloned in MCS1, in MCS2, or in both of plasmids pNZDual, pNZDualTT or pNZPolycist, normalized relative to their expression in pNZ-IRFP713 and pSD-I07, respectively. Expression of the DARPin surface display cassette was determined using flow cytometry and whole-cell ELISA, while that of IRFP was determined by measuring fluorescence. Error bars represent standard error of the mean (n = 3). The scheme of genes is depicted in the grey background.

The expression of infrared fluorescent protein (IRFP)^22^ and human IgG-binding designed ankyrin repeat protein (DARPin) I07 – surface anchor fusion^23,24^ from dual protein expression plasmids was normalized relative to their expression from pNZ8148 (pNZ-IRFP713 and pSD-I07, respectively). The absence of expression of the two model proteins from empty-plasmid backbones was confirmed. Expression of single genes located at either MCS was tested in pNZDual. It was slightly lower than that of the normalization control, with the exception of IRFP in MCS2 (Fig. 2). Concomitant expression of the two model proteins was observed from all three plasmids; however, the expression of each was again lower than that from normalization control plasmids. The expression of both proteins from pNZDual and pNZDualTT was higher when DARPin was cloned in MCS1, and IRFP in MCS2. Increase in the yield of both proteins with an additional transcription terminator downstream of MCS1 (pNZDualTT) was observed only when IRFP was cloned in MCS1, and DARPin in MCS2. Expression from pNZPolycist favored the expression of the gene in MCS1. This was particularly evident when IRFP was cloned in MCS2, resulting in its lower yield. The yield of proteins from MCS1 of pNZPolycist was generally higher than that observed from MCS1 of pNZDual or pNZDualTT.

### Construction of a single plasmid CRISPR-Cas9 system for *L. lactis*

**Figure 3:**
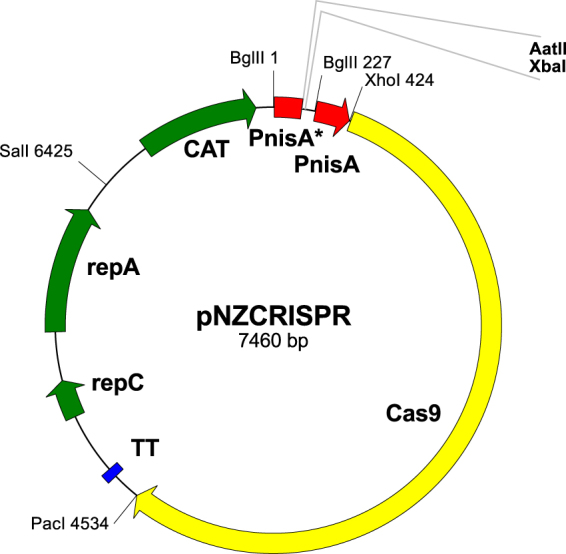
Scheme of plasmid pNZCRISPR. PnisA: nisin promoter. PnisA*: nisin promoter without ribosome binding site. Plasmid pNZCRISPRi contains dCas9 instead of Cas9 in pNZCRISPR.

Assembly of the *L. lactis* CRISPR-Cas9 plasmid pNZCRISPR was achieved in three steps. In the first, the section of pNZ8148 containing the nisin promoter and MCS was replaced by a variant of the nisin promoter lacking RBS. The AatII site was positioned immediately upstream of the transcription start site (as defined in^25^), enabling transcription of sgRNA without the addition of any further bases. Use of the AatII site led to alteration of only two bases of the nisin promoter (…TACAATGAttTCN* → …TACAATGAcgTCN*), suggesting minimal interference with promoter activity. In the second step, a second nisin promoter was cloned into pNZ8148noRBS, yielding plasmid pNZ8148noRBS_dualPnisA, and, in the third step, Cas9 gene was cloned into the latter, yielding plasmid pNZCRISPR (Fig. 3). pNZCRISPR is a functional CRISPR-Cas9 system in which a gene of choice for sgRNA can be inserted.

### Use of the CRISPR-Cas9 system against *htrA* gene in the lactococcal genome

**Figure 4:**
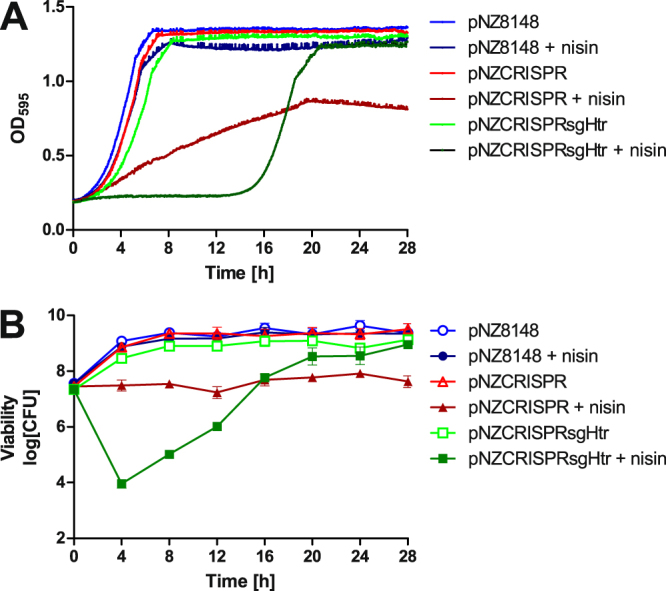
Growth curves (**A**) and viability (**B**) of *L. lactis* containing pNZ8148, pNZCRISPR or pNZCRISPRsgHtr in the presence or absence of the inducer nisin. Growth curves were measured in four repeats and representative curve is shown. Error bars represent standard deviation of the mean (n = 3).

The effectiveness of pNZCRISPRsgHtr in *L. lactis* was compared with that of the control plasmid pNZCRISPR lacking specific sgRNA, or empty plasmid pNZ8148. No difference in the growth curve of *L. lactis* containing plasmids pNZCRISPRsgHtr, pNZCRISPR or pNZ8148 was observed without the addition of nisin (Fig. 4A). The addition of nisin to pNZ8148-containing bacteria had little effect on the growth curve; growth rate decreased from 0.287 ± 0.007 to 0.240 ± 0.010. When nisin was added to pNZCRISPR-containing bacteria, the growth rate was decreased considerably, from 0.262 ± 0.005 to 0.038 ± 0.001. However, when nisin was added to pNZCRISPRsgHtr-containing bacteria, a significant extension of the lag phase was observed (15.52 h compared to 3.40 h with pNZCRISPRsgHtr-containing bacteria without nisin, or to pNZ8148- and pNZCRISPR-containing bacteria (around 2 h)). When the exponential phase finally commenced, the growth rate (0.242 ± 0.007) was very similar to that observed without the addition of nisin (0.245 ± 0.002).

Viability of the same cultures was assessed every 4 h (Fig. 4B). The viability of pNZ8148-, pNZCRISPR- or pNZCRISPRsgHtr-containing cells without nisin inducer increased for 2-logs over the period of 8 h, and then remained relatively constant. The addition of nisin had little effect on pNZ8148-containing cells, and similar increase in viability was observed as in the absence of nisin. On the contrary, the addition of nisin to pNZCRISPR-containing cells resulted in just a small increase in viability over the course of 24 h. More pronounced effect was observed by the addition of nisin to pNZCRISPRsgHtr-containing cells. Rapid, almost 4-log drop in cell viability was observed over the course of first 4 h. However, the decrease was reversed over the course of next 24 h and the final viability was similar to that observed with other plasmid-containing cultures.

### Use of the CRISPR-Cas9 system against *ermR* gene in pIAV7 plasmid

**Figure 5:**
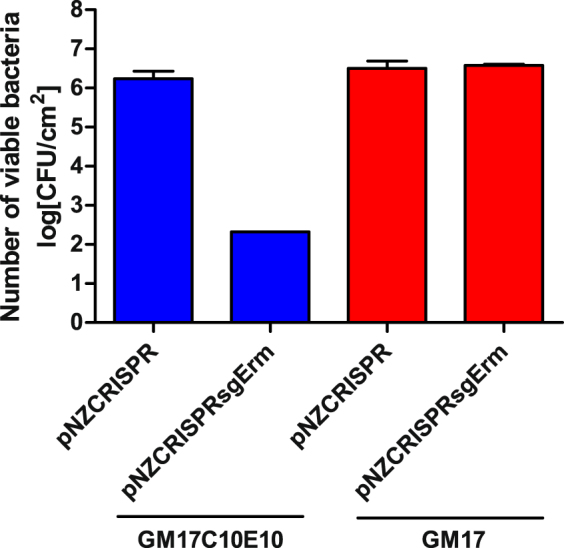
Survival of *L. lactis* containing either pNZCRISPR and pIAV7 or pNZCRISPRsgErm and pIAV7, when incubated in the presence of inducer nisin and plated on plates containing chloramphenicol and erythromycin (GM17C10E10), or on non-selective plates (GM17). Error bars represent standard error of the mean (n = 3).

The CRISPR-Cas9 system was used against the *ermR* gene in pIAV7 plasmid^26^ that carries an erythromycin resistance marker, and can therefore be used concomitantly with pNZCRISPR that carries a chloramphenicol resistance marker. By directing Cas9 to the erythromycin-resistance gene, using *ermR*-targeting sgRNA (plasmid pNZCRISPRsgErm), the plasmid pIAV7 was inactivated, resulting in a 4-log decrease in cell viability in the presence of erythromycin (Fig. 5). No decrease in viability in the presence of erythromycin was observed for control *L. lactis* co-transformed with pIAV7 and pNZCRISPR (without *ermR*-targeting sgRNA), indicating the absence of non-specific inactivation of pIAV7 (Fig. 5). Also, *L. lactis* co-transformed with pIAV7 and either pNZCRISPR (control) or pNZCRISPRsgErm exhibited no difference in survival when plated on media without antibiotics, indicating that *ermR*-targeting sgRNA is not detrimental to the cells and is not causing cleavage of the chromosome that would result in cell death (Fig. 5).

### CRISPRi mediated silencing of *upp* gene decreases *upp* mRNA transcription and prevents the toxicity of 5-fluorouracil

**Figure 6:**
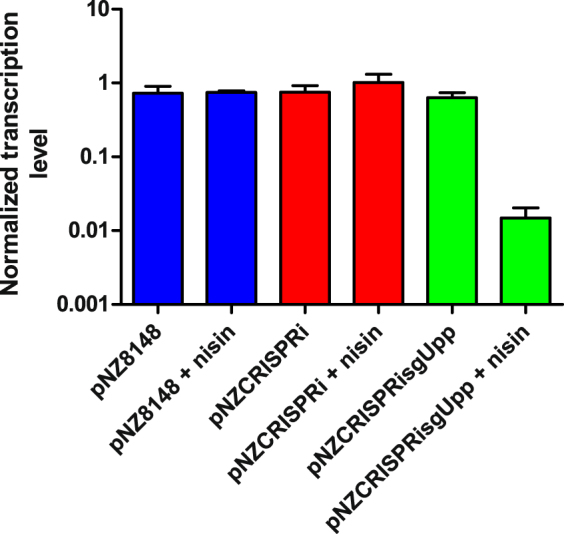
Normalized transcription level, determined by qPCR, of *upp* gene in *L. lactis* containing pNZ8148, pNZCRISPRi, or pNZCRISPRisgUpp, with or withour nisin induction. Error bars represent standard error of the mean (n = 3).

*L. lactis* containing pNZCRISPRisgUpp in the presence of inducer nisin had more than 50-fold lower relative transcriptional level of *upp* in comparison to *L. lactis* containing pNZCRISPRisgUpp without inducer, *L. lactis* containing pNZCRISPRi with or without inducer, or *L. lactis* containing pNZ8148 with or without inducer (Fig. 6). No major differences in relative transcriptional level of *upp* were observed among the latter, indicating tight control of induction and the absence of unspecific dCas9 action.

**Figure 7:**
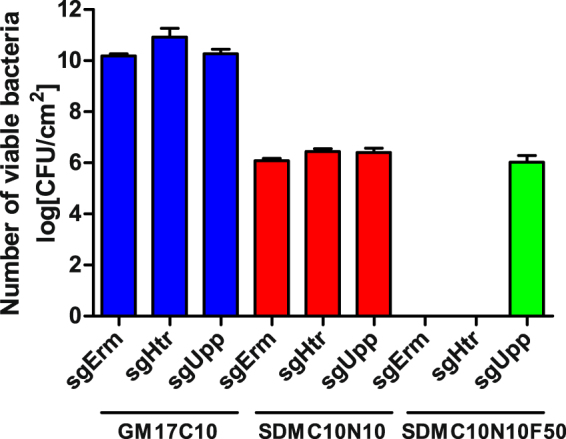
Survival of *L. lactis* transformed with pNZCRISPRsgErm (sgErm), pNZCRISPRisgHtr (sgHtr) or pNZCRISPRisgUpp (sgUpp). Each was inoculated from an overnight culture, incubated in the presence of the inducer nisin and plated on plates containing complex medium with chloramphenicol (GM17C10), semi-defined medium with chloramphenicol and nisin (SDMC10N10), or semi-defined medium with chloramphenicol, nisin and 5-fluorouracil (SDMC10N10F50). Error bars represent standard error of the mean (n = 3).

*L. lactis* containing pNZCRISPRisgUpp (transcribing sgUpp and expressing dCas9) was able to survive in the presence of 5-fluorouracil on semi-defined medium, substantiating *upp* silencing (Fig. 7). The same conditions were lethal for control bacteria without *upp* silencing that contained either pNZCRISPRisgHtr (transcribed genome-targeting sgHtr and expressed dCas9), or pNZCRISPRsgErm (transcribed sgErm and expressed Cas9). All three bacterial species exhibited similar viabilities in either GM17 or semi-defined media (SDM; Fig. 7), further confirming that the differences in survival in media containing 5-fluorouracil are the consequence of *upp* silencing.

## Discussion

In the present study we have developed new tools for genetic engineering of the industrially relevant lactic acid bacterium *L. lactis*. A series of plasmids for inducible dual protein expression in *L. lactis* were constructed, and their efficacy confirmed by expressing two model proteins. The toolbox was expanded by further modifying the plasmids to create inducible single-plasmid CRISPR-Cas9 and CRISPRi systems capable of concomitant transcription of sgRNA and expression of Cas9/dCas9. CRISPR-Cas9 and CRISPRi were used to inactivate or silence three model genes.

New NICE plasmids for protein co-expression with two PnisA promoters (pNZDual and pNZDualTT) were prepared on the basis of pNZ8148, in a manner analogous to that reported for protein co-expression in *E. coli* using the T7 promoter system^11^. Additionally, to facilitate application of the plasmids and cloning procedures, two unique multiple cloning sites (MCS1 and MCS2) were included downstream of each promoter. The original NICE plasmid, pNZ8148, contains the NcoI site that enables translational fusion with the ATG start codon. The NdeI site is also commonly used for that purpose; however it is already present in the pNZ8148 backbone. The NdeI site of the pNZ8148 plasmid was therefore removed to enable inclusion of NdeI in MCS2. An additional transcription terminator was inserted downstream of MCS1, since it has been reported that the presence of a terminator downstream of each gene increases protein yield^8^. An alternative approach to protein co-expression has been reported that uses a single promoter and multiple genes separated by ribosome binding sites (RBS), enabling polycistronic transcription^10^. The polycistronic transcription plasmid pNZPolycist (Fig. 1C) was also obtained from pNZ8148. It contains one PnisA promoter followed downstream by two MCSs, separated by the RBS sequence, AGGAGG.

Co-expression of proteins using pNZDual, pNZDualTT or pNZPolycist was examined using two model proteins previously used effectively in our research^24,27^. IRFP^22^ is an infrared fluorescent protein with an emission maximum at 713 nm that has been used for *in vivo* imaging of bacteria in mice^27^. It also enables direct measurement of fluorescence in bacterial culture. Its fluorescence intensity is directly proportional to the concentration of bacteria or to the quantity of the protein produced^27^. The DARPin surface display cassette^24^ consists of the Usp45 secretion signal^28^, the affinity scaffold DARPin I07^23^ and the AcmA surface anchor^29^. Expression of the cassette results in the surface display of DARPin I07 which has high affinity for human IgG and has been proposed as a model non-immunoglobulin scaffold binder for the surface display and delivery by LAB^24,30^. *L. lactis* co-expressing IRFP and DARPin I07 constitutes a model LAB with concomitant imaging and therapeutic capabilities. The co-expression of the two model proteins was more balanced in pNZDual than in pNZPolycist; the latter generally favored the expression of the gene immediately downstream of the promoter, in accordance with previous reports^9^. Interestingly, expression of both proteins, IRFP and DARPin cassette, was higher when the DARPin cassette was cloned in MCS1 and IRFP in MCS2, an issue that was not resolved by introduction of the additional transcription terminator (pNZDualTT). However, the latter was beneficial in the inverse configuration of genes, with IRFP in MCS1 and DARPin cassette in MCS2. This indicates that the expression level is protein-dependent and that different combinations should be tested when optimizing the co-expression conditions.

The dual protein expression plasmid pNZDual was further modified and used for constructing the single plasmid CRISPR-Cas9 system for *L. lactis*. A functional CRISPR-Cas9 system for biotechnological application requires three elements: a CRISPR array, that is transcribed to CRISPR RNA (crRNA), a complementary trans-activating RNA (tracrRNA), and a Cas9 protein that is guided by crRNA-tracrRNA hybrid and cleaves the target DNA^15^. The system can be simplified by fusing crRNA and tracrRNA into a single guide RNA (sgRNA). This results in a two-component system^31^ that, in the present case, could be developed from the two promoter backbone of pNZDual. However, direct use of pNZDual was prevented, by the presence of numerous restriction enzyme recognition sites of MCS1 and MCS2 in *cas9* gene of *Streptococcus pyogenes*. Additionally, one of the nisin promoters had to be tailored in order for sgRNA to be transcribed without subsequent translation; one of the ribosome binding sites (RBS) downstream of the promoter therefore had to be removed. This resulted in the plasmid pNZ8148noRBS that lacks RBS downstream of nisin promoter and could be applied for the inducible transcription of RNA of e. g. riboswitches or aptamers. The inducible RNA transcription plasmid pNZ8148noRBS was further developed into pNZCRISPR and pNZCRISPRi by including a second promoter and unique restriction sites XhoI and PacI that were used for the insertion of Cas9 or dCas9 genes.

The CRISPR systems were tested by targeting *htrA, ermR* and *upp* genes. The CRISPR-Cas9 system, that targets the genome of *L. lactis*, causes a double strand break in the chromosome and is lethal to the bacterial cells. Assembly of a system targeting genome encoded *htrA*, together with constitutive expression of Cas9 and transcription of sgRNA against *htrA*, would therefore be impossible. However, by using nisin-inducible expression plasmids no growth retardation could be observed without the addition of nisin. The addition of nisin to pNZCRISPR-containing cells (without sgRNA) decreased the growth rate in comparison to control plasmid pNZ8148-containing cells. This might be attributed to the metabolic burden of Cas9 overexpression with strong nisin promoter, or unspecific action of Cas9 in the absence of sgRNA, and should be taken into account in further applications of the system. The plasmid pNZCRISPRsgHtr retarded growth only in the presence of nisin which induced the expression of Cas9 and transcription of *htrA*-targeting sgRNA. The pronounced effect was observed as extension of the lag phase from 3.40 h to 15.52 h and was explained by a rapid 4-log drop in viability that could not be observed spectrophotometrically. However, the viability started to increase after the first four hours and reached the initial viability after 16 hours, when the start of the exponential phase was recorded on the growth curve. Extension of the lag time has recently been related to the efficacy of antibiotics^32^, which have consequences for the bacteria similar to those of genome-targeting Cas9. The large extension of the lag phase and increase in viability after initial drop was probably caused by a limited number of CRISPR survivals that evaded Cas9 lethality. These results therefore confirm the activity of the CRISPR-Cas9 system in *L. lactis*.

While CRISPR-Cas9-mediated targeting of the genome results in lethality for the cell, targeting of the gene located on a plasmid results in plasmid curing and its loss. The CRISPR-Cas9 system directed against *ermR* gene on pIAV7 plasmid decreased the survival of cells that were co-transformed with pIAV7 and pNZCRISPRsgErm in the presence of erythromycin. The decrease in survival indicates CRISPR-Cas9-mediated inactivation of the pIAV7 plasmid that contains the *ermR* gene. The extent of decrease (4-log) is in accordance with those reported previously (3–5-log) ^14^ and those observed with *htrA* targeting.

An alternative application of CRISPR, termed CRISPRi, is used for precise, targeted genome regulation and represents a valid alternative to RNAi^15^. This regulation is achieved by using mutated, nuclease-inactivated Cas9 (dead Cas9 or dCas9). This can be guided by specific sgRNA to target genes and promoters where it physically prevents initiation of transcription and elongation^33^. CRISPRi activity in *L. lactis* was demonstrated by silencing *upp* gene encoding uracil phosphoribosyltransferase that decreased relative transcription of *upp* mRNA more than 50-fold. Uracil phosphoribosyltransferase is responsible for conversion of 5-fluorouracil into nucleotide and its subsequent toxicity. By silencing the *upp* gene with pNZCRISPRisgUpp the cells became resistant to 5-fluorouracil, in accordance with previous reports in which *upp* gene was inactivated^34,35^.

To summarize, duplication of the nisin promoter enabled balanced, inducible expression of two model proteins in *L. lactis*, thus constituting a new tool for recombinant protein expression in this organism. A similar strategy resulted in a single plasmid CRISPR-Cas9 system that can be used, among other possible applications, for plasmid curing or for CRISPRi-mediated gene regulation in *L. lactis*, although limitations, such as curing of CRISPR plasmid and silencing of more than one gene, will have to be overcome in future studies. The new plasmid series that was developed in the present research will nevertheless advance the genetic engineering techniques of the economically important lactic acid bacterium *L. lactis* and facilitate studies of its physiology.

## Materials and Methods

### Bacterial strains, media and culture conditions

*L. lactis* NZ9000 was grown at 30 °C in M-17 medium (Merck) supplemented with 0.5% glucose (GM-17) without aeration. To maintain selection pressure, 10 µg/mL of chloramphenicol, or 10 µg/mL of erythromycin, or both, was added when required. 15.5 µg/mL biliverdin HCl (Sigma Aldrich) was added for the expression of IRFP. Semi-defined medium (SDM)^36^ was used for studying resistance to 5-fluorouracil (added at 50 µg/mL). *E. coli* strain DH5α was grown at 37 °C with aeration in LB medium supplemented with 100 µg/mL ampicillin.

### General cloning procedures

Either Taq DNA Polymerase (Thermo Fisher), KOD DNA Polymerase (Merck Millipore), or Phusion Hot Start II DNA Polymerase (Thermo Fisher) was used for gene amplifications according to the manufacturer’s instructions. Primers were from IDT and restriction enzymes from Thermo Scientific (Fast Digest). NucleoSpin Gel and PCR Clean-up (Macherey and Nagel) was used for isolating DNA fragments from agarose gels. T4 DNA ligase was from New England Biolabs. PCR amplicons were routinely cloned to pGEM-T Easy (Promega) and sub-cloned to lactococcal plasmids. Plasmid DNA was isolated with NucleoSpin Plasmid (Macherey and Nagel), with an additional lysozyme treatment step for *L. lactis*. Lactococci were transformed with electroporation using a Gene Pulser II apparatus (Biorad) according to the MoBiTec GmbH (Goettingen, Germany) instructions. All plasmids were sequenced by GATC (Constance). All primers and plasmids are listed in Supplementary Table S1.

### Introduction of a second nisin promoter and a multiple cloning site to pNZ8148 to create an inducible dual protein expression plasmid and a single plasmid CRISPR-Cas9 system

The NdeI site was removed from pNZ8148 by site directed mutagenesis. pNZ8148 was amplified using NZ_NdeI-del_F/ NZ_NdeI-del_R primer pair and KOD DNA polymerase. Amplicon was agarose gel purified and digested with NdeI to remove the plasmid template. Digested DNA was transformed in *L. lactis* NZ9000. Colonies containing plasmid with mutated NdeI (pNZ8148m) were identified with restriction digest and used for plasmid isolation.

Nisin promoter (PnisA) was amplified from pNZ8148 using MCS2-F-Xba/ MCS2-R-Hind and Taq polymerase. Amplicon was digested, using XbaI/HindIII restriction enzymes, and ligated into equally prepared pNZ8148m, yielding pNZDual (Fig. 1A). Transcription terminator was amplified from pNZ8148 with the TT-Xba-F/TT-Spe-R primer pair. The amplicon was digested with XbaI/SpeI (BcuI) restriction enzymes (that form compatible overhangs) and cloned into XbaI-digested pNZDual, yielding pNZDualTT (Fig. 1B) with a single XbaI site. RBS in fusion with MCS2 was amplified with partially overlapping primers RBS_MCS2_ Xba_F/ RBS_MCS2_ Hind_R, digested with XbaI/HindIII restriction enzymes and ligated into equally prepared pNZ8148m, resulting in pNZPolycist (Fig. 1C).

A single plasmid CRISPR-Cas9 system was established in three steps. First, RBS downstream of PnisA was removed and MCS was altered by replacing the PnisA-MCS, via BglII/XbaI restriction sites, with a PCR amplicon that was obtained with Nis-woRBS-BglII-F/Nis-woRBS-AatXba-R primers and pNZ8148 template. The isolated plasmid was termed pNZ8148noRBS. Secondly, another PnisA amplicon was obtained with MCS2-Xba-F2/MCS2-XhoPacHind-R primer pair, using pNZ8148 as a template. Amplicon was digested with XbaI/HindIII restriction enzymes and cloned into equally prepared pNZ8148noRBS, resulting in plasmid pNZ8148noRBS_dualPnisA. Thirdly, Cas9 gene was amplified from pVPL3004, using Cas-Xho-F/Cas-Pac-R primer pair and Phusion DNA polymerase, digested with XhoI/PacI and ligated into equally prepared pNZ8148noRBS_dualPnisA, thereby yielding pNZCRISPR (Fig. 3).

CRISPR interference (CRISPRi) plasmid pNZCRISPRi was prepared by replacing Cas9 gene with dCas9 gene. The latter was amplified from pcDNA-dCas9 using primers Cas-Xho-F and Cas-Pac-R.

### Cloning of model genes and single guide RNAs (sgRNAs)

IRFP and surface display cassette of DARPin I07 were established as model proteins in our previous work^24,27^. IRFP gene was cut from pNZ-IRFP713 with NcoI/XbaI and ligated into equally digested pNZDual (MCS1) or pNZPolycist (MCS1). For cloning into MCS2 of pNZDual or pNZPolycist, IRFP gene was amplified from pNZ-IRFP713, using IRFP-F-Nde/IRFP-R-Xho primer pair, and digested using NdeI and XhoI restriction enzymes. Similarly, DARPin I07 surface display cassette was cut from pSD_I07 with NcoI/XbaI and ligated into equally digested pNZDual (MCS1) or pNZPolycist (MCS1). For cloning into MCS2 of pNZDual or pNZPolycist, DARPin I07 surface display cassette was amplified from pSD_I07, using USP-F-Nde/A3b-R-Xho primer pair, and digested using NdeI and XhoI restriction enzymes. IRFP gene and DARPin I07 surface display cassette were cloned in either MCS1, MCS2, or in both, of pNZDual and pNZPolycist, in different combinations, resulting in plasmids pNZDual_DARPin1, pNZDual_DARPin2, pNZDual_IRFP1, pNZDual_IRFP2, pNZDual_DARPin1_IRFP2, pNZDual_IRFP1_DARPin2, pNZPolycist_DARPin1_IRFP2 and pNZPolycist_IRFP1_DARPin2 (Supplementary Table S1). IRFP gene and DARPin I07 surface display cassette were also cloned in pNZDualTT, resulting in pNZDualTT_DARPin1_IRFP2 and pNZDualTT_IRFP1_DARPin2.

The generic sequence of sgRNA^37^ begins with the 20 bp protospacer sequence that can be altered in accordance with the target. Appropriate protospacers were selected manually on the basis of the target sequences by searching the available protospacer adjacent motifs (PAMs). Single guide RNA against erythromycin resistance gene (sgErm) was assembled from two partially overlapping primers, sg_erm3-F and sg_erm3-R, using Taq DNA polymerase, and then cloned to pGEM-T Easy, yielding pGEMsgErm. The latter was digested with AatII and XbaI restriction enzymes and ligated to equally prepared pNZCRISPR, yielding pNZCRISPRsgErm. pGEMsgErm was used as a template for the amplification of other sgRNAs, using a sense primer with target-specific 5′-region (sg_htrA_F, sg_upp_F) and sgRNA-R antisense primer. Single guide RNAs against genes for housekeeping protease HtrA (sgHtr) and uracil phosphoribosyltransferase (sgUpp) were amplified in a similar manner. sgHtr was cloned in pNZCRISPR and pNZCRISPRi, yielding pNZCRISPRsgHtr and pNZCRISPRisgHtr, respectively. sgUpp was obtained, by manually searching PAM sites, to target the 100 bp sequence downstream of the *upp* gene transcription initiation site. sgUpp was cloned in pNZCRISPRi, yielding pNZCRISPRisgUpp.

### Expression of model proteins IRFP and DARPin surface display cassette

Overnight cultures of *L. lactis* NZ9000 harbouring either pNZ8148m, pNZDual, pNZDualTT, pNZPolycist, pNZ-IRFP713, pSD_I07, pNZDual_DARPin1, pNZDual_DARPin2, pNZDual_IRFP1, pNZDual_IRFP2, pNZDual_DARPin1_IRFP2, pNZDual_IRFP1_DARPin2, pNZDualTT_DARPin1_IRFP2, pNZDualTT_IRFP1_DARPin2, pNZPolycist_DARPin1_IRFP2, pNZPolycist_IRFP1_DARPin2, pNZDualTT_DARPin1_IRFP2 or pNZDualTT_IRFP1_DARPin2 plasmids were diluted (1:100) in 10 mL (or 100 mL) of fresh GM-17 medium and grown to an optical density A_600_ = 0.80. Expression of proteins was induced with 25 ng/mL nisin (Fluka AG, Buchs, Switzerland). After three hours incubation, 1 mL of culture was stored at 4 °C for flow cytometric analysis and the remaining cell culture centrifuged at 5,000 × g for 10 min. The cell pellet was resuspended in PBS to an optical density A600 = 1.0 and stored at 4 °C for whole-cell ELISA.

### Whole-cell ELISA

The whole-cell enzyme-linked immunosorbent assay (ELISA) was carried out as described^24^. For testing the DARPin surface display, 750 μL of *L. lactis* cell suspension in PBS with optical density A_600_ = 1.0 was centrifuged (5,000 × g, 5 min, 4 °C) and washed twice with 500 μL PBS. Cells were then resuspended in 200 μL of fluorescein isothiocyanate (FITC)-conjugated human IgG (Jackson ImmunoResearch, West Grove, USA; diluted 1: 500 in PBS), and incubated 1 h at room temperature (RT) with gentle shaking. Cells were then washed twice with PBS and resuspended in 200 μL of peroxidase-conjugated mouse anti-human IgG (Jackson ImmunoResearch, West Grove, USA) solution diluted 1:2500 in PBS. After 1 h incubation at RT with gentle shaking, cells were washed, first with PBS and then with substrate buffer (150 mM Na_2_HPO_4_, 50 mM citric acid, pH 6.0). The cells were then resuspended in 1 mL of substrate buffer, and 100 μL of the appropriate dilutions (1:5 and 1:25) in substrate buffer were loaded on a microtiter plate. 100 μL of 3,3′,5,5′-tetramethylbenzidine (TMB) substrate (Sigma-Aldrich, Steinheim, Germany) was added and the reaction stopped after 15 min by adding 50 μL of 2 M sulphuric acid. Absorbances were read at 450 nm using an Infinite M1000 (Tecan, Salzburg, Austria).

### Flow cytometry

For flow cytometry, 10 μL of cell culture in stationary phase was added to 500 μL of Tris-buffered saline (TBS; 50 mM Tris-HCl, 150 mM NaCl, pH 7.5) and centrifuged for 5 min at 5,000 × g at 4 °C. The pellet was resuspended in 500 μL of TBS and 1 μL of FITC-conjugated human IgG antibody (Jackson ImmunoResearch, West Grove, USA) then added. After 2 h incubation at RT with constant shaking at 100 rpm, cells were washed three times with 200 μL 0.1% TBST and finally resuspended in 500 μL TBS. Samples were analysed with a flow cytometer (FACS Calibur; Becton Dickinson, Franklin Lakes, USA) using excitation at 488 nm and emission at 530 nm in the FL1 channel. The geometric mean fluorescence intensity (MFI) of at least 20,000 bacterial cells in the appropriate gate was measured. The average of at least three independent experiments was considered.

### Fluorescence of IRFP

Aliquots of cell cultures (200 µl) were transferred to black, flat-bottom 96-well plates (Greiner). Fluorescence was measured on an Infinite M1000 microplate reader (Tecan), with excitation/emission at 690 nm/713 nm. Nine measurements were made per single well and averaged. The measurements were made in three technical replicates. Fluorescence intensity was normalized to a cell density with OD_600_ = 1.0.

### Effects of inducing Cas9 expression and sgHtr transcription on the growth of *L. lactis*

Overnight cultures of *L. lactis* NZ9000 harbouring pNZ8148, pNZCRISPR or pNZCRISPRsgHtr were diluted (1:100) in 200 µL of fresh GM17 growth medium with or without 10 ng/mL nisin in a 96-well microplate. The plate was sealed with a sealing film and incubated in a Sunrise microplate reader (Tecan) at 30 °C for 32 hours. Absorbance was measured at 595 nm every 2 min. The plate was shaken for 10 sec before each measurement. Each culture was grown in quadruplicate. The growth rate and lag phase of the growth curves were analysed with DMFit 3.5 software using the model of Baranyi and Roberts^38^. Aliquots of the same cultures were incubated separately at 30 °C. Ten-fold dilutions of the cultures in PBS were plated, in 4 h intervals, on GM-17 plates containing 10 µg/mL chloramphenicol using the drop-plate method^39^. The colonies were counted after 2 days of incubation at 30 °C.

### Effects of induction of Cas9 expression and sgErm transcription on the growth of *L. lactis*/pIAV7

*L. lactis* NZ9000 was sequentially transformed, firstly with pIAV7 (yielding *L. lactis*/pIAV7) and, secondly, with pNZCRISPR or pNZCRISPRsgErm (yielding *L. lactis*/pIAV7/pNZCRISPR and *L. lactis*/pIAV7/pNZCRISPRsgErm). Overnight cultures of *L. lactis* NZ9000 harbouring pIAV7^26^/pNZCRISPR or pIAV7/pNZCRISPRsgErm, grown in GM17 medium containing 10 µg/mL chloramphenicol and 10 µg/mL erythromycin, were diluted (1:50) in 5 mL of fresh GM17 medium containing 10 µg/mL chloramphenicol. After reaching an OD_600_ between 0.2 and 0.5, the cultures were diluted (1:20) into fresh GM17 containing 10 µg/mL chloramphenicol and 25 ng/mL nisin and grown for 5 h. Each culture was grown in triplicate. Ten-fold dilutions of the cultures in PBS were plated on GM-17 plates containing 10 µg/mL chloramphenicol and 10 µg/mL erythromycin using the drop-plate method^39^. The colonies were counted after 2 days of incubation at 30 °C.

### Effects of induction of dCas9 expression and sgUpp transcription on the growth of *L. lactis* in the presence of 5-fluorouracil

Overnight cultures of *L. lactis* NZ9000 harbouring pNZCRISPRsgErm, pNZCRISPRisgHtr or pNZCRISPRisgUpp were diluted (1:50) in 5 mL of fresh GM17 medium containing 10 µg/mL chloramphenicol. After reaching A_600_ = 0.80, the cultures were induced with 25 ng/mL nisin and grown for 3 h. Each culture was grown in triplicate. Ten-fold dilutions of the cultures in PBS were plated on either GM-17 plates containing 10 µg/mL chloramphenicol, SDM plates containing 10 µg/mL chloramphenicol and 10 ng/mL nisin, or SDM plates containing 10 µg/mL chloramphenicol, 25 ng/mL nisin and 50 µg/mL 5-fluorouracil, using the drop-plate method^39^ and colonies were counted after 2 days of incubation at 30 °C.

### Real-time quantitative PCR (qPCR)

Overnight cultures of *L. lactis* NZ9000 harbouring pNZCRISPRi, pNZCRISPRisgUpp, or pNZ8148 were diluted (1:100) in 10 mL of fresh GM17 medium containing 10 µg/mL chloramphenicol and, optionally, 10 ng/mL nisin. After reaching A_600_ = 0.50, the cells were harvested and total RNA was isolated using High Pure RNA Isolation kit (Roche Applied Science, Germany) and quantified using NanoDrop Spectrophotometer (Thermo Scientific, USA). First strand cDNA was generated from 1 µg of RNA using High Capacity cDNA Reverse Transcription kit with RNase inhibitor (Life Technologies, USA) and random primers according to the manufacturer’s instructions. qPCR analysis was performed for the gene of interest (*upp*) and two reference genes (*recA, ldh*) in each sample using FastStart Universal SYBR Green Master (Roche Applied Science, Germany) on a StepOnePlus Real-time PCR system (Applied Biosystems, USA). Primer pairs were designed using Primer3 software and are shown in Supplementary Table S1. Cycling conditions were set at 95 °C for 10 min, followed by 45 cycles at 95 °C for 10 s and 62 °C for 35 s. Cycling was completed by a melting curve analysis. Control reactions without template were included in the assays. PCR efficiencies were at least 90% for all primer pairs and a single melting peak was observed for each primer pair. Relative gene expression was calculated upon normalization to two reference genes (*recA, ldh*), corrected for primer-specific PCR efficiency and considering error propagation as described previously^40^.

## Electronic supplementary material

Supplementary Table S1
