# Supplementary material for: Single plasmid systems for inducible dual protein expression and for CRISPR-Cas9/CRISPRi gene regulation in lactic acid bacterium Lactococcus lactis
Source: Sci Rep. 2018 Jan 17;8:1009. doi: 10.1038/s41598-018-19402-1 (PMC5772564; doi:10.1038/s41598-018-19402-1)
Supplement: Supplementary file 1 — Supplementary Table S1 [file 41598_2018_19402_MOESM1_ESM.pdf]

Supplementary Information

Single plasmid tools for dual protein expression and CRISPR/Cas9 in lactic acid bacterium *Lactococcus lactis*:

Aleš Berlec[a,*], Katja Škrlec[a,b], Janja Kocjan[a,d], Maria Olenic[a,c], Borut Štrukelj[a,d]

[a] Department of Biotechnology, Jožef Stefan Institute, Jamova 39, SI-1000, Ljubljana, Slovenia
[b] Graduate School of Biomedicine, Faculty of Medicine, University of Ljubljana, SI-1000, Ljubljana, Slovenia
[c] Faculty of Pharmacy, Charles University in Prague, 500 05, Hradec Králové, Czech Republic
[d] Faculty of Pharmacy, University of Ljubljana, Aškerčeva 7, SI-1000, Ljubljana, Slovenia

Supplementary Table S1: Primers and plasmids used in the study.

**Primer or plasmid**	**Primer sequence or plasmid feature**	**Reference**
Primers		
NZ_NdeI-del_F	CTTGATTATATCAAAGGTTCTTATGAATATTTGACTCATG	This study
NZ_NdeI-del_R	CATGAGTCAAATATTCATAAGAACCTTTGATATAATCAAG	This study
MCS2-F-Xba	TTCTAGAAATGTCACTAACCTGCCC	This study
MCS2-R-Hind	TAAGCTTCTCGAGGAGCTCACATGTCATATGGAGTGCCTCCTTATAATTTATTTTG	This study
pNZ-F1498	TAACAATAGAAAGCGTTAGG	This study
pNZ-F3072	ATGAGATAATGCCGACTG	This study
USP-F-Nde	TCATATGGCTAAAAAAAAGATTATCTCAG	This study
A3b-R-Xho	TCTCGAGTTATTCTTCCATTACACCAATTTG	This study
IRFP-F-Nde	TCATATGGCTGAGGGATCTGTAG	This study
IRFP-R-Xho	TCTCGAGTTATTCTTCCATTACACCAATTTGC	This study
A3b-R-Xho2	TTATTTCTCGAGTTATTTTATTCGTAGATACTGACC	This study
USP-F-Nde2	TTATTTCATATGGCTAAAAAAAAGATTATCTCAG	This study
RBS_MCS2_ Xba_F	ATCTAGAATTATAAGGAGGCACTCCATATGACATGTGAGC	This study
RBS_MCS2_ Hind_R	TAAGCTTCTCGAGGAGCTCACATGTCATATGGAGTGCCTC	This study
TT-Xba-F	AAAATATCTAGAGATAAAGCAATTACTGATATTGC	This study
TT-Spe-R	TCACAAACTAGTAATAAACGCTAAAACGTCTC	This study
Nis-woRBS-BglII-F	AAATTAAGATCTAGTCTTATAACTATACTGAC	This study
Nis-woRBS-AatXba-R	AAATTATCTAGAATCGATGACGTCATTGTATCTAACAAACTTCAG	This study
MCS2-Xba-F2	AAATAATCTAGAAATGTCACTAACCTGCCC	This study
MCS2-XhoPacHind-R	ATTATTAAGCTTTTAATTAAACCGGTCTCGAGTGCCTCCTTATAATTTATTTTG	This study
Cas-Xho-F	AATTTACTCGAGATGGATAAGAAATACTCAATAGGC	This study
Cas-Pac-R	ATTTGCTTAATTAATCAGTCACCTCCTAGCTG	This study
sg_erm3-F	GACGTC**TTGGATATTCACCGAACACT**GTTTTAGAGCTAGAAATAGCAAGTTA	This study

	AAATAAGGCTAGTCCGTTATC[1]	
sg_erm3-R	TCTAGAAAAGCACCGACTCGGTGCCACTTTTTCAAGTTGATAACGGACTAGCCTTATTTTAACTTGC	This study
sg_htrA_F	AGACGTC**ACGGATGCTGCCATTAACCC**GTTTTAGAGCTAGAAATAGCAAG[1]	This study
sg_upp_F	GACGTC**GAGAGTTTGTGTTGAATCAA**GTTTTAGAGCTAGAAATAGCAAG[1]	This study
sgRNA-R	TCTAGAAAAGCACCGACTCG	This study
Cas_seq1	GATTTAGATAATTTATTGGCGC	This study
Cas_seq2	ATCGAAAAGTAACCGTTAAGC	This study
Cas_seq3	GATTATGATGTCGATCACATTG	This study
upp97	GTTGACGAAATCGGAATGCT	This study
upp291	CACACGAGCAGCTGGAATTA	This study
ldh38	GTGACGGTGCTGTAGGTTC	[1]
ldh159	ATGAGAAAGGTCTTCTGCATCC	[1]
rec912	ATCTGGTGCTTGGTTTGCTT	This study
rec1028	GCCCGAACTTTGTGGTCTATT	This study
Plasmids		
pNZ8148	pSH71 derivative, PnisA, CmR, nisin-controlled expression	[2-4]
pIAV7	pWV01 derivative, broad range, EmR, *lacZ*, T1T2	[5]
pVPL3004	pNZ9530 derivative, EmR, *nisRK* replaced with the tracrRNA, cas9 and CRISPR array	[6]
pNZ8148m	pNZ8148ΔNdeI	This study
pNZDual	pNZ8148 derivative, two nisin promoters, MCS1, MCS2	This study
pNZPolycist	pNZ8148 derivative, nisin promoter, MCS1, MCS2, separated by RBS	This study
pNZDualTT	pNZ8148 derivative, two nisin promoters, MCS1, MCS2, two TT	This study
pNZ-IRFP713	pNZ8148 containing *irfp713*	[7]
pSD-I07	pNZ8148 containing gene fusion of SP_{Usp45}, DARPin I07 and cA	[8]
pNZDual_DARPin1	pNZDual containing fusion of SP_{Usp45}, DARPin I07 and cA in MCS1	This study
pNZDual_DARPin2	pNZDual containing fusion of SP_{Usp45}, DARPin I07 and cA in MCS2	This study
pNZDual_IRFP1	pNZDual containing *irfp713* in MCS1	This study
pNZDual_IRFP2	pNZDual containing *irfp713* in MCS2	This study
pNZDual_DARPin1_IRFP2	pNZDual containing fusion of SP_{Usp45}, DARPin I07 and cA in MCS1 and *irfp713* in MCS2	This study
pNZDual_IRFP1_DARPin2	pNZDual containing *irfp713* in MCS1 and fusion of SP_{Usp45}, DARPin I07 and cA in MCS2	This study
pNZPolycist_DARPin1_IRFP2	pNZPolycist containing fusion of SP_{Usp45}, DARPin I07 and cA in MCS1 and *irfp713* in MCS2	This study
pNZPolycist_IRFP1_DARPin2	pNZPolycist containing *irfp713* in MCS1 and fusion of SP_{Usp45}, DARPin I07 and cA in MCS2	This study
pNZDualTT_DARPin1_IRFP2	pNZDualTT containing fusion of SP_{Usp45}, DARPin I07 and cA in MCS1 and *irfp713* in MCS2	This study
pNZDualTT_IRFP1_DARPin2	pNZDualTT containing *irfp713* in MCS1 and fusion of SP_{Usp45}, DARPin I07 and cA in MCS2	This study
pNZ8148noRBS	pNZ8148 derivative without RBS after nisin promoter	This study
pNZ8148noRBS_dualPnisA	pNZ8148noRBS derivative, second nisin promoters	This study

pGEMsgHtr	pGEM-T Easy containing sgHtr	This study
pGEMsgErm	pGEM-T Easy containing sgErm	This study
pGEMsgUpp	pGEM-T Easy containing sgUpp	This study
pNZCRISPR	pNZ8148noRBS_dualPnisA derivative with *cas9* in MCS2	This study
pNZCRISPRsgErm	pNZCRISPR with sgErm in MCS1	This study
pNZCRISPRsgHtr	pNZCRISPR with sgHtr in MCS1	This study
pNZCRISPRi	pNZ8148noRBS_dualPnisA derivative with *dcas9* in MCS2	This study
pcDNA-dCAS9	pcDNA3.1 derivative with *dcas9*	[9]
pNZCRISPRisgUpp	pNZCRISPRi with sgUpp in MCS1	This study

[1]Protospacer is shown in bold.

References:

1 Friedrich, U. & Lenke, J. Improved enumeration of lactic acid bacteria in mesophilic dairy starter cultures by using multiplex quantitative real-time PCR and flow cytometry-fluorescence in situ hybridization. *Applied and environmental microbiology* **72**, 4163-4171, doi:10.1128/AEM.02283-05 (2006).

2 de Ruyter, P. G., Kuipers, O. P. & de Vos, W. M. Controlled gene expression systems for Lactococcus lactis with the food-grade inducer nisin. *Applied and environmental microbiology* **62**, 3662-3667 (1996).

3 Kuipers, O. P., de Ruyter, P. G. G. A., Kleerebezem, M. & de Vos, W. M. Quorum sensing-controlled gene expression in lactic acid bacteria. *J Biotechnol* **64**, 15-21 (1998).

4 Mierau, I. & Kleerebezem, M. 10 years of the nisin-controlled gene expression system (NICE) in Lactococcus lactis. *Applied microbiology and biotechnology* **68**, 705-717, doi:10.1007/s00253-005-0107-6 (2005).

5 Perez-Arellano, I., Zuniga, M. & Perez-Martinez, G. Construction of compatible wide-host-range shuttle vectors for lactic acid bacteria and Escherichia coli. *Plasmid* **46**, 106-116, doi:10.1006/plas.2001.1531 (2001).

6 Oh, J. H. & van Pijkeren, J. P. CRISPR-Cas9-assisted recombineering in Lactobacillus reuteri. *Nucleic acids research* **42**, e131, doi:10.1093/nar/gku623 (2014).

7 Berlec, A., Zavrsnik, J., Butinar, M., Turk, B. & Strukelj, B. In vivo imaging of Lactococcus lactis, Lactobacillus plantarum and Escherichia coli expressing infrared fluorescent protein in mice. *Microbial cell factories* **14**, 181, doi:10.1186/s12934-015-0376-4 (2015).

8 Zadravec, P., Strukelj, B. & Berlec, A. Improvement of LysM-mediated surface display of designed ankyrin repeat proteins (DARPins) in recombinant and nonrecombinant strains of Lactococcus lactis and Lactobacillus Species. *Applied and environmental microbiology* **81**, 2098-2106, doi:10.1128/AEM.03694-14 (2015).

9 Perez-Pinera, P. *et al.* RNA-guided gene activation by CRISPR-Cas9-based transcription factors. *Nature methods* **10**, 973-976, doi:10.1038/nmeth.2600 (2013).
